# Therapeutic Activity of Green Tea Epigallocatechin-3-Gallate on Metabolic Diseases and Non-Alcoholic Fatty Liver Diseases: The Current Updates

**DOI:** 10.3390/nu15133022

**Published:** 2023-07-03

**Authors:** Armachius James, Ke Wang, Yousheng Wang

**Affiliations:** 1 Key Laboratory of Geriatric Nutrition and Health, Ministry of Education, Beijing Technology and Business University, Beijing 100048, China; armachiuss@gmail.com (A.J.); wangke9509@163.com (K.W.); 2 Tanzania Agricultural Research Institute (TARI), Makutupora Center, Dodoma P.O. Box 1676, Tanzania; 3 Rizhao Huawei Institute of Comprehensive Health Industries, Shandong Keepfit Biotech. Co., Ltd., Rizhao 276800, China

**Keywords:** green tea, EGCG, metabolic disease, hyperglycemia, hyperuricemia, non-alcoholic fatty liver disease

## Abstract

Green tea polyphenols have numerous functions including antioxidation and modulation of various cellular proteins and are thus beneficial against metabolic diseases including obesity, type 2 diabetes, cardiovascular and non-alcoholic fatty liver diseases, and their comorbidities. Epigallocatechin-3-gallate (EGCG) is the most abundant polyphenol in green tea and is attributed to antioxidant and free radical scavenging activities, and the likelihood of targeting multiple metabolic pathways. It has been shown to exhibit anti-obesity, anti-inflammatory, anti-diabetic, anti-arteriosclerotic, and weight-reducing effects in humans. Worldwide, the incidences of metabolic diseases have been escalating across all age groups in modern society. Therefore, EGCG is being increasingly investigated to address the problems. This review presents the current updates on the effects of EGCG on metabolic diseases, and highlights evidence related to its safety. Collectively, this review brings more evidence for therapeutic application and further studies on EGCG and its derivatives to alleviate metabolic diseases and non-alcoholic fatty liver diseases.

## 1. Introduction

Green tea is an important beverage in most Asian countries and is popularly enjoyed by the Chinese and the Japanese. It is produced from *Camellia sinensis* fresh leaves. Green tea contains antioxidant compounds, vitamins, carbohydrates, proteins, minerals, chlorophyll, and polyphenols, which provide health benefits [1,2]. Green tea polyphenols have strong antioxidants and display the ability to quench and scavenge reactive oxygen species (ROS). The most prominent effects of green tea on human health are mainly attributed to catechins including epicatechin, epigallocatechin, epicatechin gallate, and epigallocatechin-3-gallate (EGCG) [3].

**Figure 1:**
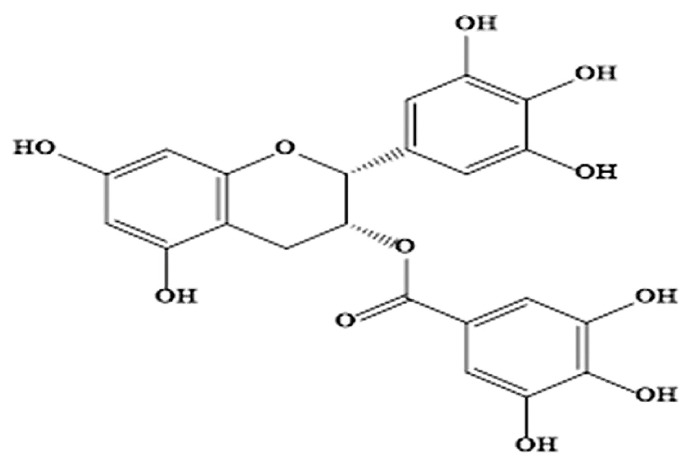
Epigallocatechin-3-gallate (EGCG): a catechin ester of epigallocatechin and gallic acid with three aromatic rings linked by a pyran ring, which contributes to its functional benefits.

EGCG is the most efficacious compound and is key to the biological activities of green tea (Figure 1). Green tea contains a high concentration of active EGCG, which accounts for about 50% of total polyphenols in tea leaves [4]. The EGCG, a flavone-3-ol polyphenol is the most promising bioactive phytochemical due to its strong antioxidant activity and prebiotic function [5,6]. EGCG is mainly absorbed in the intestine, and gut microbiota plays a critical role prior to absorption as microbiota can metabolize large molecular tea polyphenols to bioactive and bioactive microbial metabolites [7]. Oral administration of EGCG showed good bioavailability as analyzed in the plasma when administered to healthy individuals after overnight fasting [8]. In a randomized crossover experiment, Van Amelsvoort et al. [9] reported an insignificant amount of EGCG excreted in urine when healthy individuals were administered with EGCG, which suggests that EGCG is transported to the liver or metabolized by the gut microbiota to other potential metabolites. Indeed, it has been established that EGCG can be detected in plasma after ingestion and its metabolites are identified in bile, plasma, and urine.

Metabolic syndrome or syndrome X is indicated by elevated blood sugar, elevated blood cholesterol, hyperlipidemia or dyslipidemia, excessive body fat, insulin resistance, visceral obesity, and arterial hypertension. Moreover, metabolic disease patients have been diagnosed with hyperuricemia, while serum urate stands as the biomarker for metabolic syndrome [10,11]. Consequently, elevated serum urate is closely associated with gout, coronary heart disease, hypertension, non-alcoholic fatty liver disease (NAFLD), and type 2 diabetes [12,13]. The modern world is characterized by highly processed high-sugar and high-fat diets together with a sedentary lifestyle exposing the population across all age groups to an unprecedented increase in metabolic diseases. The increasing incidences of metabolic diseases and associated comorbidities have become a hot spot of research globally. From obesity, type 2 diabetes, cardiovascular diseases, kidney diseases, and gout to NAFLD, EGCG has been examined for its alleviating effect on the diseases. EGCG has been reported to have a uric acid-lowering effect on metabolic disease patients, particularly through reduced uric acid production or increased uric acid excretion. Notably, the EGCG anti-hyperuricemia effect is mediated by xanthine oxidase (XOD) inhibition, an enzyme that catalyzes hypoxanthine to xanthine to uric acid [14]. Moreover, EGCG increases energy expenditure, improves fat oxidation, and reduces respiratory quotient, thereby influencing body mass index (BMI) and total body fat, and is a preventive agent for obesity and oxidative stress [15]. The ability of EGCG to bind several biological molecules, influence enzyme activities, and signal transduction pathways may explain its health benefits.

On the other hand, EGCG may be harmful in high doses when taken in the extract forms, and reports on toxicity are emerging. High doses of EGCG not only cause cytotoxicity in vitro but also may result in hepatoxicity, nephrotoxicity, and gastrointestinal disorders such as vomiting and diarrhea. Therefore, this review aims to provide current knowledge on the potential activity of EGCG as a component of green tea or extract supplements in the prevention and alleviation of metabolic syndrome, metabolic diseases, and NALFD.

## 2. The Role of EGCG on Metabolic Syndrome

Metabolic syndrome includes a group of risk factors: elevated blood sugar, insulin resistance, excessive body fat, visceral obesity, elevated blood cholesterol, hyperlipidemia, and arterial hypertension, which has become one of the major public health challenges worldwide [16,17]. The development of one of these conditions increases the risk of obesity, type 2 diabetes, hypertension, and renal and cardiovascular diseases (CVD) [17,18]. Abdominal obesity and insulin resistance have gained increased attention as the core manifestation of metabolic syndrome. Other abnormalities contributing to metabolic syndrome include chronic inflammatory and prothrombotic states, NAFLD, and sleep apnea [19]. In addition, high serum urate or hyperuricemia is a suggested metabolic syndrome indicator and a risk factor for the progression of metabolic diseases [11,20].

A patient with metabolic syndrome is in constant inflammation due to the associated concentration of chemokines, adipokines, and pro-inflammatory cytokines [21,22]. At the same time, metabolic syndrome-related chronic inflammations are implicated in a delayed and inferior immune response, with increased activation of immunosuppressive macrophages which exacerbate metabolic dysfunction [23,24]. Additionally, both humoral and cellular immune memory are impaired weakening the adaptive response of the immune system to diseases. The inflammatory markers of metabolic syndrome include increased interleukin IL-1, 6, and 8, tumor necrosis factor-α (TNF-α), resistin, white blood cell count, and C-creative protein as well as a decreased adiponectin [25,26,27]. Studies have revealed that some inflammatory cytokines, such as IL-1β and IL-18 play a crucial role in the development of arteriosclerotic plaques in patients with metabolic syndrome [28,29]. Moreover, hyperglycemia contributes to increased glucose levels in the endothelial cells, which favors the oxidative degradation of glucose metabolites with consequent oxidative stress. Costa et al. [30] conducted a systematic review and revealed that metabolic syndrome is a risk factor for the progression and prognosis of coronavirus disease 2019 (COVID-19) and elaborated that patients with metabolic disorders may face a higher risk of infection, thus complicated treatment. Overall, integrated and proper functioning metabolic homeostasis is paramount to innate immune responses.

**Figure 2:**
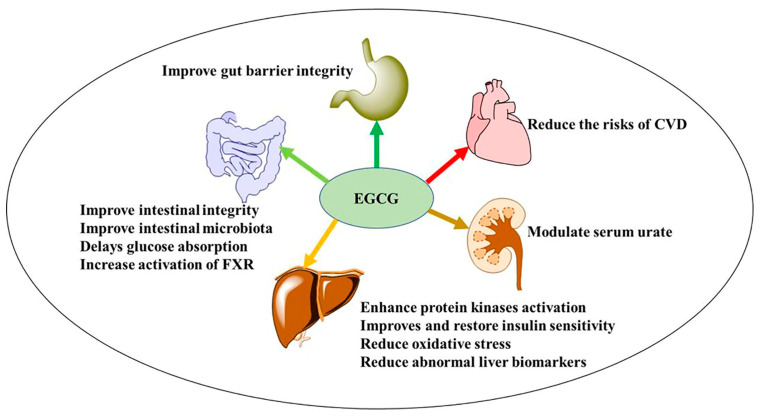
EGCG and metabolic syndrome, target the functioning of the gastrointestinal tract, the liver, the kidneys, and the heart. CVD: cardiovascular diseases and FXR: farnesoid X receptor.

The increasing risk and prevalence of metabolic syndrome demand therapeutic food-based treatment intervention. Although, the fundamental approach is lifestyle changes to reduce or remove the underlying problems, weight loss, increased physical activities, drug treatment, and a healthy diet could alleviate metabolic syndrome. Thus, green tea EGCG forms a food-based approach and a disease-preventive supplement, as it may aid in the reduction of metabolic syndrome and the onset of age-related non-communicable diseases (Figure 2). Several clinical studies have associated green tea EGCG consumption with a significant reduction in body weight, body mass index (BMI), and abdominal fat [27]. The pro-antioxidant properties of EGCG suppress gene and protein expression of adenosine monophosphate-activated protein kinases (AMPK) and transcription factors involved in adipogenesis and lipogenesis alleviating insulin sensitivity, thus leading to a reduction in body weight [31]. Recent studies suggest that EGCG exerts its beneficial effect by modulating mitochondrial functions impacting mitochondrial biogenesis, bioenergetic control in adenosine triphosphate (ATP) production, alteration of the cell cycle, and mitochondrial-related apoptosis [32,33]. In addition, EGCG appears to have a beneficial effect on the gut microbiota as increases the number of beneficial species of *Bifidobacterium*, thereby improving the energy metabolism. Simultaneously, EGCG stimulates enzymes involved in lipolysis and modulation of serum urate as well as copious pathways (Table 1). Therefore, EGCG is a promising therapy for weight management, BMI, and waist circumference reduction, as well as improving lipid metabolism.

### 2.1. Effects of EGCG on Insulin Resistance and High Blood Pressure

Insulin resistance is identified as an impaired biological response to insulin stimulation of the target tissues, prominently in the muscles, liver, and adipose tissue [51]. It impairs sensitivity to insulin-mediated glucose disposal, resulting in a compensatory increase in pancreatic β-cell insulin production to maintain normal blood glucose levels. Consequently, it results in a cluster of abnormalities including hyperinsulinemia, hyperglycemia, dyslipidemia, visceral adiposity, obesity, hyperuricemia, hypertension, endothelial dysfunction, and elevated inflammatory markers [52].

Insulin increases glucose uptake in the muscles and liver and inhibits hepatic gluconeogenesis and lipolysis. However, insulin resistance impairs insulin-mediated inhibition of lipolysis in adipose tissues leading to increased circulating lipids, which further inhibit the antilipolytic effect of insulin [51]. Non-esterified fatty acids or free fatty acids (FFAs) inhibit protein kinase activation in the muscles leading to reduced glucose uptake. Conversely, FFAs increase the activation of protein kinases in the liver, promoting hepatic gluconeogenesis and stimulating adipose lipogenesis as well as lipolysis [53,54,55]. Thus, higher levels of circulating FFAs directly affect the liver and muscle metabolism, and further aggravate insulin resistance [56]. Overall, the progression of insulin resistance may lead to metabolic syndrome, polycystic ovary syndrome, non-alcoholic fatty liver disease (NAFLD), type 2 diabetes, sleep apnea, and certain forms of cancer.

The anti-insulin resistance and glucose homeostasis effects of EGCG have been consistently described. Yan et al. [57] revealed that green tea catechins significantly decreased glucose levels and improved glucose tolerance in the animal experiment. Green tea EGCG reduced ROS in adipocytes, attenuated dexamethasone, and TNF-α as a result of ROS, and increased glucose uptake ability hence alleviating adipose insulin resistance [57]. Fasting serum glucose, insulin levels, and insulin resistance were reduced significantly in obese hypertensive patients following the uptake of green tea extract in the randomized double-blind, placebo control clinical trial study [58]. Similarly, Liu et al. [44] conducted a randomized double-blind, placebo control clinical trial experiment involving 92 subjects with type 2 diabetes and lipid abnormalities; found that green tea extract (GTE) significantly alleviated insulin resistance, increased glucagon-like peptide-1 and high-density lipoprotein (HDL) levels, and decreased triglycerides levels. At the same time, EGCG ameliorated insulin resistance by upregulating and increasing phosphorylation of the insulin receptor substrate-1 (IRS-1), which is essential for the stimulation of glucose uptake in response to insulin [59]. For instance, EGCG reversed high glucose- and glucosamine-induced insulin resistance in SH-SY5Y neuronal cells by improving the oxidized cellular status and mitochondrial function [59]. Similarly, a study that employed a GTE in mice, showed that EGCG attenuated insulin resistance induced by a high-fat diet [47]. Additionally, EGCG was shown to improve glucose tolerance in mice [60]. According to Lee et al. [61] green tea-derived products such as extracts and water-soluble polysaccharides exhibit hypoglycemic effects as they caused delayed intestinal absorption of glucose. The hypoglycemic mechanism of EGCG has been contributed by its inhibitory effect on α-glucosidase activity, enhancement of glucose uptake, and promotion of glucose transporter-4 (GLUT4) translocation to the plasma membrane through a phosphatidylinositide-3-kinase/activated protein kinase B (PI3K/AKT) signaling pathway in skeletal muscle cells [62,63]. When EGCG uptake was combined with regular exercise in overweight or obese postmenopausal women, it resulted in reduced plasma glucose concentration in subjects with impaired glucose tolerance [64]. Collectively, green tea EGCG alleviates insulin resistance, increases glucose uptake, and lowers blood glucose, which are important for glucose homeostasis.

### 2.2. Effects of EGCG on Adipose Mass, Blood Cholesterol, and Triglycerides

Several studies suggest that EGCG can decrease energy and food intake, lipogenesis as well as preadipocyte differentiation and proliferation, while increasing lipolysis, and fat oxidation. Green tea EGCG was revealed to reduce tissue and blood lipid accumulation in the FFAs-induced human liver hepatocellular carcinoma cell line (HepG2) via activation of the AMPK pathway [43]. Consequently, AMPK activation shifts some FFAs toward oxidation, away from lipid and triglycerides storage, and suppresses hepatic gluconeogenesis, which is implicated in the reduction of adipose mass and body weight.

Findings from a systematic review by Asbaghi et al. [65] revealed that, supplementing >800 mg GTE/day for eight or more weeks significantly improved lipid profile by reducing serum triglycerides and total cholesterol concentrations in patients with type 2 diabetes. Similarly, the consumption of green tea EGCG significantly lowered low-density lipoprotein (LDL) as well as total cholesterol levels in normal weight and obese individuals [66]. Moreover, supplementing EGCG for four to eight weeks to patients with obesity reduced plasma triglycerides and serum kisspeptin levels [67].

A study involving healthy Japanese women revealed that elevated plasma and urinary concentration of green tea catechins was associated with improved plasma lipid profile [68]. Randomized double-blind placebo-controlled clinical trials involving obese women in Taiwan, reported a significant decrease in total cholesterol, LDL, and triglyceride, and increased levels of HDL as well as plasma adiponectin in groups administered with GTE for 12 weeks [69,70]. Furthermore, a combination of EGCG and caffeine produced a synergistic effect on gut microbiota: increasing *Bifidobacterium* count and fecal short-chain fatty acid (SCFAs) levels and enhanced fecal bile acids excretion in experimental rats [71]. At the same time, the combination effect increased the expression of hepatic G-coupled protein receptor 1 and activation of intestinal farnesoid X receptor (FXR). The activation of intestinal and hepatic FXR induces endocrine hormone fibroblast growth factor 15 (FGF15) and small intestine heterodimer partner production, which collectively inhibits hepatic bile acid biosynthesis via signaling cascades [71]. A randomized double-blind parallel placebo-controlled clinical trial showed that administering 400, 600, or 800 g EGCG (depending on body weight) for 12 months in men with Down syndrome resulted in weight loss, reduced body fat, and improved lipid profile [72]. In addition, Choi. et al. [60] revealed that EGCG regulates lipid catabolism through AMPK-mediated mechanisms increasing lipolysis and suppressing lipogenesis in the adipocytes. Therefore, EGCG reduces visceral adiposity by activating autophagy and lipolysis in white adipose tissue through an AMPK-mediated mechanism.

### 2.3. Effects of EGCG on Hyperuricemia and Uric Acid Metabolism

Uric acid is the final catabolic product of the enzymatic degradation of purines as well as other dietary components and can scavenge ROS, thus protecting the erythrocytes membrane from oxidation in humans [73]. Hyperuricemia is considered a metabolic disease, while elevated serum uric acid is the metabolic disease biomarker [20]. Hyperuricemia induces oxidative stress and endothelial dysfunction, resulting in the development of a series of diseases including insulin resistance, type 2 diabetes, coronary artery diseases, chronic kidney diseases, kidney stone, and gout, thus becoming a metabolic disease that threatens human health [13,74]. Moreover, hyperuricemia has been reported to involve in the manifestation of NAFLD. Maintaining serum urate levels below 7 and 6 mg/dL in men and women, respectively, is clinically important for the prevention of hyperuricemia, type 2 diabetes, cerebrovascular, cardiovascular diseases, and gout [75].

At present drugs such as allopurinol, oxypurinol, febuxostat, and topiroxostat, which are xanthine oxidase inhibitors, and the recombinant uricase (rasburicase), uricosuric agent (probenecid) are currently used to treat hyperuricemia [76,77]. However, these drugs have side effects such as causing uric acid stones, liver and kidney stones, liver damage, and/or may lead to hypersensitivity reactions, which may not be tolerated by the patient [77,78]. Of interest, EGCG has been studied to have a uric acid-lowering effect. Thus, can be used to manage or develop nutraceutical drugs for hyperuricemia and alleviate metabolic diseases.

In animal studies using mice, tea ranging from green, yellow, black, or dark tea extract significantly increased uric acid excretion by upregulating the expression of uric acid secretion transporters ABCG2, organic anion transporter 1 (OTA1), organic anion transporter 3 (OTA3) and organic cation transporter 1 (OCT1), and by downregulating the expression of uric acid reabsorption transporter; urate transporter 1 (URAT1) and glucose transporter 9 (GLUT9) in the kidney [79,80]. Likewise, Li et al. [14] reported that EGCG significantly promoted the expression of OAT1 and downregulated the expression of GLUT9 in renal tissues of hyperuricemia rats. At the same time, tea extract significantly lowers serum urate levels through the inhibition of XOD and ADA to produce uric acid [38]. Moreover, interventions using tea extract revealed that tea components upregulate the expression of the intestinal ABCG2 protein and alleviated hyperuricemia by modulating the gut microbiota. The study of Sang et al. [80] reports yellow tea to be the best in alleviating hyperuricemia in mice and indicated 50% EGCG oral bioavailability.

Using human normal liver cell line HL-7702 (L-02), Wu et al. [81] revealed that tea extract limit uric acid production via inhibition of XOD activity, with green tea showing the strongest inhibitory activity followed by yellow, oolong, white, black, and dark tea. Zhang et al. [37], using spectroscopic and computer simulation methods, found that EGCG at a concentration of 0.13 mmol/L inhibited 80% XOD activity by binding to the vicinity of flavine adenine dinucleotide (FAD) in XOD, hindering the entry of the substrate. In xanthine-stimulated BRL 3A rat liver cells, EGCG significantly reduced uric acid levels in vitro. Additionally, it was revealed that EGCG significantly reduced serum uric acid and inhibited XOD activity in rats treated with potassium oxonate [14].

A randomized cross-over study in Japan revealed a significant increase in the excretion of uric acid and uric acid precursor (xanthine and hypoxanthine) in the group of healthy men receiving distilled spirit (Shōchū) with catechin-enriched green tea [82]. Similarly, Jatuworapruk et al. [83] reported the hypouricemic effect of green tea in healthy individuals, that serum uric acid decreased with decreased uric acid clearance after two weeks of the randomized study. The studies on healthy individuals reflected the idea of green tea extract components particularly EGCG to inhibit XOD activity and thus reduce the production of uric acid and increase the excretion of uric acid precursor (xanthine and hypoxanthine) with urine.

## 3. Effect of EGCG on Metabolic Diseases

### 3.1. Obesity

Obesity is a major public health burden that leads to chronic inflammation and metabolic disorders in both peripheral tissues and the central nervous system. Obesity is mainly caused by an energy imbalance between calorie intake and utilization, which results in adipose tissue dysfunction with adipocyte hypertrophy, excessive accumulation of adipose tissue, and to an extent that impairs physical health and psychological well-being [84]. Obesity comprises several metabolic alterations accompanied by a state of chronic inflammations and an increased oxidative state that contributes to the development of an array of health complications. It increases the risk of insulin resistance, high blood glucose levels, dyslipidemia (high triglyceride and cholesterol levels), age-related cognitive impairment, arteriosclerosis progression, and peripheral inflammation.

Obesity is one of the major risk factors for type 2 diabetes, hypertension, and CVD. Worldwide, >35% of adults are considered to be obese, and in some countries, obesity prevalence exceeds 40% [85,86]. Additionally, obesity is estimated to reach 18% and 21% in men and women, respectively, by 2025 [71,84]. Interestingly, obesity had previously been identified as a risk factor for viral infections due to its influence on the immune response. For instance, during the 2009 H1N1 outbreak, obese patients presented severe complications [87,88]. Likewise, during the recent COVID-19 pandemic, obese patients exhibited a high rate of complications and a need for hospitalization [89,90].

The intake of EGCG appears to be a promising strategy for the prevention and management of obesity and its complications. The study by Chatree et al. [67] showed that EGCG supplementation for eight weeks significantly decreased fasting plasma triglyceride levels, blood pressure, and serum kisspeptin levels in humans. A meta-analysis of randomized controlled clinical trials on the influence of green tea intake on obesity indices in humans revealed a significant reduction in body weight, BMI, and a reduced waist circumference at a dosage of <500 mg of green tea per day for 12 weeks [91]. In a randomized placebo-controlled trial involving 60 healthy Japanese people in Japan, administration of a combined dose of 146 mg EGCG in green tea and citrus polyphenol (178 mg α-glucosyl hesperidin) for 12 weeks prevented weight gain and reduced the BMI [92]. Similarly, the obesity-related indicators including triglycerides levels, visceral and body fat percentage, as well as blood LDL/HDL ratio decreased in the <50 years group [92].

In the mice experiment, it was revealed that EGCG significantly ameliorated insulin resistance and cognitive disorder by upregulating IRS-1 and extracellular signal-regulated kinases (ERK)/cAMP response element-binding protein (CREB)/brain-derived neurotrophic factor (BDNF) signaling pathways [59]. Zhou et al. [93] showed that GTE inhibits the release of the inflammatory cytokine TNF-α, IL-1β and IL-6 in palmitic acid-induced BV-2 microglial cells by suppressing the JAK2/signal transducer and activator of transcription-3 (STAT3) signaling pathway. Furthermore, the animal experiment that recruited obese rats treated with GTE showed a significant reduction in obesity indicators through AMPK activation, restored insulin sensitivity, and stimulated fatty acid oxidation in the plasma and liver [94].

### 3.2. Type 2 Diabetes Mellitus

Diabetes is a group of metabolic diseases characterized by hyperglycemia, resulting from defects in insulin secretion, insulin action, or both. A predisposition to glucose intolerance depends on various factors that share an ability to stress the glucose homeostasis profile, with the current explosion of sedentary lifestyles, obesity, and insulin resistance being the major causes of type 2 diabetes [95]. Failure of insulin to induce an adequate response of the target tissues and the level of insulin receptors contributes to metabolic abnormalities in glucose homeostasis [96]. The skeletal muscles, adipose tissues, liver, pancreatic β-cell, brain, and vascular endothelium are the major insulin targets. Acquired defects in glucose homeostasis cause blood glucose levels to rise to a range of intolerable impaired glucose tolerance. The rise of blood glucose causes additional deterioration of beta-cell function along with further insulin resistance and elevated hepatic glucose; subsequently, blood glucose rises to full-blown type 2 diabetes. In addition to suppressing the patient’s immunity, type 2 diabetes can cause metabolic dysfunction that directly affects homeostasis and is associated with a range of debilitating complications such as CVD, chronic kidney diseases, and visual disability or blindness [55,96]. Thus, the global prevalence of type 2 diabetes is estimated at 6.28%, which is equivalent to 462 million individuals [97], and is projected to reach 700 million individuals by 2045 [98].

Several drugs, both insulin and non-insulin formulations, have been in use to manage and alleviate diabetes. They include different formulations of exogenous insulin, insulin simulants (glimepiride, glipizide, repaglinide, nateglinide, sitagliptin, and saxagliptin), α-glucosidase inhibitors (acarbose and miglitol), glucagon-like peptide-1 receptor agonistics (sulfonylureas, meglitinides, exenatide, and semaglutide) and insulin sensitivity enhancers (metformin and rosiglitazone) among others [99,100]. However, the drugs may display adverse effects and complications in patients such as risks of hypoglycemia, weight gain, headache, genitourinary tract infection, gastrointestinal tract disturbances, and cardiovascular events including heart failure [101,102]. Additionally, the use of the existing drugs requires prior diagnosis adding up to the healthcare costs to manage the disease. In addition, diabetic patients present complicated individualized treatment strategies due to associated side effects, contraindications, and underlying comorbidities, thus a major challenge to the health care systems. With this in mind, improving metabolic control to normal glucose homeostasis through the intake of EGCG as a supplement or part of green tea can greatly benefit a long-term, sustainable, and safe intervention.

Green tea EGCG has been demonstrated to improve insulin sensitivity and glycemic control, and significantly decrease serum triglycerides and total cholesterol levels following a long-term supplementation at ≤800 mg/day [65]. In addition, green tea EGCG decreased triglycerides and significantly increased HDL and glucagon-like peptide 1 levels in a randomized double-blinded placebo control clinical trial involving patients with type 2 diabetes and lipid abnormalities for 16 weeks in Taiwan [44]. According to Liu et al. [103] EGCG can reverse pancreatic β-cell damage or apoptosis and enhance glucose-stimulated insulin secretion or insulin sensitivity by decreasing the expression of microRNA (miR-16-5p), which targets the anti-apoptotic β-cell lymphoma-2 (BCL-2). The cell surface protein 67-kDa laminin receptor was revealed to act as the sensor for EGCG inducing the production of nitric oxide in the endothelial cells while downregulating the production of inducible nitric oxide synthase (iNOS) enzymes, thereby mediating the beneficial effect of EGCG through cyclic guanosine monophosphate (cGMP) dependent pathway and cellular nitric oxide production [104,105,106]. Therefore, EGCG improves endothelial function and reduced oxidative stress through decreased nitric oxide production and decreased expression of iNOS. Likewise, the EGCG-derived autoxidation products have been revealed to improve insulin sensitivity through suppression of liver-derived secretory selenocysteine-containing selenoprotein P (SELENOP) implicated to cause insulin resistance [107]. For instance, theasinensin A an oxidation product of EGCG displayed high cellular uptake on HePG2 cells as well as higher antioxidant capacity compared to the monomer EGCG [108]. Thus, EGCG can be effective in controlling hyperglycemia and alleviating the complications of diabetes by improving insulin sensitivity and reducing the risk factors for type 2 diabetes. In addition, a study by Hadi et al. [109] which involved 50 diabetic patients concluded that consuming 300 mg EGCG/day for eight weeks significantly decreases fasting blood glucose, body weight, and the high-sensitive C-reactive proteins, thus alleviating type 2 diabetes. A systematic review and meta-analysis reported that consumption of green tea for more than eight weeks significantly decreased body weight, BMI, and body fat in diabetic patients [39].

A high-fat diet may activate the nucleotide-binding oligomerization domain-like receptor protein 3 (NLRP3) inflammasome in macrophages and participate in immune dysfunction leading to chronic inflammation and insulin resistance. The pro-inflammatory cytokines interleukin IL-1β and IL-18 play important roles in inflammatory diseases and type 2 diabetes. They are regulated by the inflammasomes, which process inactive pro- IL-1β and pro-IL-18 proteins into active IL-1β and IL-18 proteins, respectively [110]. Inflammasomes are the key targets for EGCG. In the same way, EGCG may exert anti-inflammatory effects against NLRP3, which enhances insulin signaling [111]. According to Zhan et al. [112] administration of EGCG in a type 2 diabetic mouse model provided an anti-inflammasome effect and improved glucose tolerance in vivo. Therefore, the suppression of NLRP3 inflammasome-mediated inflammation is a possible mode of action of EGCG for alleviation and treatment of type 2 diabetes.

### 3.3. Cardiovascular Diseases

Cardiovascular diseases (CVD) are implicated in the abnormal function of the heart and blood vessels. It includes coronary heart diseases, peripheral arterial diseases, congenital heart diseases, rheumatic heart diseases, and cerebrovascular diseases [113]. Arteriosclerosis is a disease of arteries that is caused by endothelial dysfunction, inflammatory vascular cells, and lipid accumulation. Plaque which is mostly composed of lipid, calcified, fibrous, fibrolipidic, or necrotic cores is the culprit that causes arteriosclerosis, and can partially or completely block the blood flow in the arteries [45]. Cardiovascular diseases occur as a complication and are comorbid with other metabolic diseases. For instance, approximately 34.8% of CVD occurs concurrently with type 2 diabetes across countries as revealed in the cross-sectional study on CVD across continents [114]. The prevalence of CVD in South Asian countries is as high as 49.6% among adult individuals [115] and contributes to a third of overall death in the Americas [116]. Globally, CVD has contributed to about 17.4 and 17.8 million deaths in 2012 and 2017, respectively [117,118].

There are a number of studies assessing green tea EGCG consumption with respect to CVD. Consumption of green tea and administration of GTE or EGCG has been reviewed to reduce the risks and mortality rate owing to CVD [119]. The therapeutic effects of EGCG on CVD are associated with the inhibition of LDL cholesterol, inhibition of NF-κB, reduction of plasma glucose and glycated hemoglobin levels, inhibition of myeloperoxidase activity, reduction of inflammatory markers and inhibition of ROS generation. For instance, when EGCG uptake was combined with regular exercise in overweight or obese postmenopausal women reduced the resting heart rate [64]. Lange [120] presented a review of population-based and epidemiological studies in Japan, North America, and Europe, and reported that habitual green tea intake of two to six cups a day was associated with reduced risks of CVD. In a clinical trial in Iran, Mozaffari-Khosravi et al. [121] reported a significant decrease in systolic and diastolic blood pressure in mildly hypertensive type 2 diabetic individuals who consumed three glasses of green or sour tea daily for four consecutive weeks. Likewise, Peng et al. [122] performed a meta-analysis of randomized controlled trials, and suggested that consumption of green tea or a low dose of green tea polyphenols significantly decreases systolic and diastolic blood pressure by 1.98 and 1.92 mmHg, respectively, in humans. In a randomized double-blind placebo-controlled cross-over experiment, it was revealed that a single dose of 300 mg EGCG alleviated endothelial function and improved arterial-mediated dilation in patients with coronary arterial diseases, but no significant effect on administering 150 mg of EGCG (twice a day) for two weeks [123].

In animal experiments, rats were fed a diet containing 2 and 4 g/kg GTE with added salt (35 g/kg) to induce hypertension for 42 days; green tea indicated beneficial effects on blood pressure, markers of inflammation (TNF-α), and serum antioxidants status [124]. Similarly, EGCG was shown to attenuated salt-induced hypertension and renal injury in rats after six weeks of oral administration [125]. Ocular hypertensive patients were administered with EGCG for three months in a randomized, placebo-controlled, double-blind, cross-over clinical trial, which suggested that EGCG favorably influences inner retinal function in the eyes with early to moderately advanced glaucomatous damage, although the observed effect was small [126]. Additionally, supplementing green tea extract for three months in obese hypertensive patients significantly reduced the risks of blood pressure such as insulin resistance, inflammation and oxidative stress [58]. The reported effect may be linked to increased insulin sensitivity and suppression of leucocytes adhesion to the endothelium and transmigration through inhibition of transcription factor NF-κB-mediated production of cytokines and adhesion molecules, in both vascular endothelial and inflammatory cells [58,127]. For instance, EGCG chelates metal ions to form an inactivated complex that reduces the catalytic effects of metal ions in the oxidation reaction and effectively removes surplus active free radicals from the body, thus reducing oxidative vascular endothelium damage and the possibility of thrombosis. In contrast, a study involving elderly women and men aged ≥80 years in China revealed that green tea intake was associated with a 38% increase in the risk of developing hypertension in men, although it had no impact on women [128].

## 4. Effects of EGCG on Non-Alcoholic Fatty Liver Disease

Non-alcoholic fatty liver disease (NAFLD) is the predominant hepatic disorder worldwide affecting about 25% of the general population, which is estimated at one billion people worldwide [129,130]. Relatively, the prevalence of NAFLD in China, European countries, Japan, and the United States of America (USA) were reported at 17.6%, 17.9–25.4%, 17.9%, and 26.3%, respectively [130].

The NAFLD describes a spectrum of progressive liver conditions ranging from relatively benign liver steatosis with inflammation and advancing to non-alcoholic steatohepatitis (NASH), fibrosis, and cirrhosis [46,131]. The NASH features are indicated as fatty hepatocytes and inflammatory cell infiltrates in association with increased activation of hepatic NF-κB, which exacerbates liver injury. It is the necessary stage of NAFLD for the development of simple steatosis to cirrhosis and hepatocellular carcinoma. Recent studies have highlighted that iron loading contributes to liver damage, whereas the accumulation of free cholesterol can exacerbate NASH. While the etiology of NAFLD is unclear: genetic factors, lifestyle, ageing, and environmental factors are implicated [132]. It is generally considered as the liver component of metabolic syndrome which is associated with insulin resistance as the main pathogenetic mechanism to trigger NASH. It presents high degree of comorbidities with obesity, type 2 diabetes, and hypertension [133,134]. For instance, the metabolic associated NAFLD affects about 50.7% of obese or overweight adults globally [129]. The longitudinal cohort studies conducted in Beijing, China concluded that hyperuricemia precedes NAFLD and contributes to the development of the disease [135]. Additionally, Li et al. [136] revealed a significant association of hyperuricemia with NAFLD in a cross-sectional study in Ningbo, China. Thus, lowering serum uric acid levels and alleviating metabolic syndrome may prevent NAFLD as described in previous sections.

As the treatment options are limited and there are no effective pharmacological treatments for NAFLD, dietary approaches have been emphasized to manage NASH risks. Various studies have revealed that EGCG targets and activate the cellular AMPK pathway as well as insulin receptor substrate-1 (IRS-1) attenuating insulin resistance [94,137]. The activation of AMPK leads to increased fatty acid oxidation in the liver, and simultaneously inhibits hepatic lipogenesis and cholesterol synthesis. Subsequently, the activated AMPK pathway reduce the activity of enzymes involved in fatty deposits and triglyceride accumulation in the liver, thus alleviating NAFLD [138].

**Figure 3:**
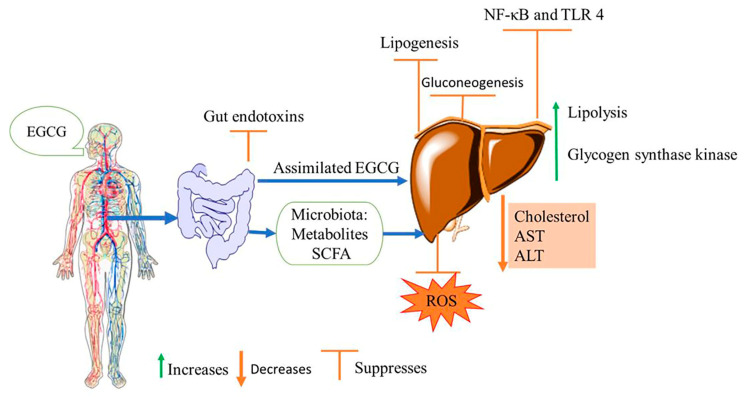
EGCG targets various pathways and metabolic processes to alleviate non-alcoholic fatty liver disease (NAFLD): SCFA: shorty chain fatty acids, ROS: reactive oxygen species, AST: aspartate aminotransferase, ALT: alanine aminotransaminase, NF-κB: nuclear factor kappa B, TLR 4: toll-like receptor 4.

The ability of EGCG to attenuate intracellular redox alterations and anti-inflammatory bioactivity responses downstream of NF-κB activation from extracellular receptors has been studied. EGCG exerts its effect indirectly through gut microbiota-derived metabolites, which limits NF-κB activation and NASH-associated liver injuries [137]. Increasing evidence suggests that EGCG may prevent and mitigate NAFLD through antioxidant activity, inhibition of endotoxins, and restoring redox homeostasis (Figure 3). The GTE or EGCG protects against NAFLD and reduces liver steatosis by reducing hepatic oxidative stress and endotoxins toll-like receptor-4 nuclear factor κB (TLR4/NF-κB) inflammation [47,139]. The EGCG benefits are linked, at least in part, to alleviating gut microbiota and improved gut barrier integrity, which limits endotoxins translocation and absorption. In addition, the protective effect against NASH has been linked to certain enzymes from the microbiome and microbiota beneficial short chain fatty acids [132,140]. According to the cross-sectional data of adult individuals from the 2009–2014 United States National Health and Nutrition Examination Survey, consumption of green tea was associated with reduced odds of having one or more abnormal liver biomarkers, namely bilirubin, alkaline phosphatase, gamma-glutamyl transferase, aspartate aminotransferase (AST), and serum alanine aminotransferase (ALT) [141]. Similar reduction of transaminases (ALT and AST) were reported in the animal model when diabetic mice were administered with EGCG [104].

## 5. Safety Implications of EGCG

The increasing incidences of metabolic diseases have promoted the increased use of food supplements and therapeutic agents including EGCG. Recently, EGCG has become one of the most studied green tea catechins due to their associated health-promoting benefits. As EGCG comes as a component of green tea, intake of green tea is considered as safe in the range of historical use in China and Japan despite high consumption levels. However, EGCG extracts safety implications have to be scrutinized and communicated as higher doses are achievable in the context of dietary supplements, particularly for weight loss formulations. A number of studies have reported events of liver toxicity either caused by green tea extracts or ingestion of EGCG supplements. The acceptable daily intake (ADI) for a 70 kg adult human was reported to be 322 mg EGCG per day [142]. The no observed adverse effect level (NOAEL) was reported to be 600 mg per day and the European Union Food Safety Authority (EFSA) indicates an intake of equal or above 800 mg EGCG a day could lead to human liver damage as indicated by elevated transaminases [142,143]. A review by Dekant et al. [144] reported no liver toxicity observed after the intake of EGCG below 600 mg EGCG per person a day with green tea infusion or tea GTE-based beverages. Recently, a randomized prospective cohort study involving 39 women (18 ≤ age ≤ 40 years) recommended a dose of 720 mg EGCG (for at least a month) alone or in combination with uterine fibroids management drugs to be tolerable without associated liver toxicity [145].

Moreover, it was demonstrated that EGCG induced liver toxicity as the function of dose, administration route, and treatment period in the animal experiments. In the animal model, subcutaneous injection of EGCG at a dose rate of 500 mg EGCG/kg body weight per day resulted in liver toxicity and 75% of experimental mice died after the first injection. The oral gavage dose at 200 mg EGCG/kg body weight in lactating mice was recorded as NOAEL, however the same dose via subcutaneous injection induced liver cell necrosis and renal tubule damage [146]. Intraperitoneal injection of 100 mg EGCG/kg per day for four consecutive days induce mice renal toxicity as indicated by elevated serum cystatin C and neutrophil gelatinase-associated lipocalin and inflammatory markers, and caused 60% mortality in streptozotocin-induced diabetic mice [147]. Moreover, an increase in nicotinamide dinucleotide phosphate (NADPH) oxidase was observed, which potentiated the production of ROS and exacerbated oxidative stress in diabetic mice injected with EGCG [147]. The daily tolerance dose for 14 consecutive days in mice was established at 21.1 or 67.8 mg EGCG/kg intraperitoneal injection and oral administration, respectively [148]. Assessing genotoxicity in mice, up to 50 and 1200 mg EGCG/kg per day by intravenous injection or oral gavage, respectively, was regarded as safe [149].

On the other hand, the chemical structure of EGCG makes it susceptible to autooxidation degradation, which may have implications regarding toxicity. Under normal physiological conditions, EGCG can be auto-oxidized to o-quinone through non-enzymatic dehydrogenation of the phenolic hydroxyl groups, which are further oxidized by oxygen to yield superoxide radicals [150]. The peroxide radicals further function as oxidants of EGCG to form o-quinone and hydrogen peroxide (H_2_O_2_), subsequently generating ROS [108]. As a consequence, oxidative stress occurs as the ROS level exceeds cellular antioxidant capacity. The prooxidant effect of EGCG and ROS generation might further display cellular or DNA-damaging activities.

Overall, an individualized safe intake level of EGCG as an extract or supplement is recommended to check on the label and calculate the intake amount as the presented ADI stands for a 70 kg body weight adult individual. Additionally, further clinical studies are recommended to clarify toxicity levels of EGCG intake in humans as most of the presented findings are based on animal experiments.

## 6. Conclusions

The safety of EGCG is well documented in animal and clinical studies as to the established acceptable daily intake (ADI) of 322 mg/day. However, intake of an amount higher than 800 mg EGCG a day may cause liver injuries. Moreover, different methods of preparing a cup of green tea, tea extract, EGCG purity, and associated catechin compounds or experimental designs applied in different studies might have contributed to some reported discrepancies in the activity of EGCG on metabolic diseases and NAFLD. Therefore, further studies to understand signaling pathways and molecular events associated with EGCG in alleviating metabolic diseases and NAFLD are recommended.

Bringing it all together, EGCG can be useful to alleviate metabolic diseases and their related malaise. Lastly, long-term use of EGCG either alone or in combination with conventional therapies for the prevention, management, and treatment of metabolic diseases and non-alcoholic fatty liver diseases remains to be an area for further research.

## Figures and Tables

**Table 1:** Molecular targets of EGCG for the prevention and therapeutic effect on metabolic syndrome/diseases.

Molecular Mechanism	Molecular Targets	References
Antioxidation	Protect against metal-induced oxidation by chelating metal ions Cell sulfur metabolism	[34]
	ROS scavenging including superoxide anion and hydroxyl radicals	[35,36]
Uric acid metabolism	Inhibitory activity on xanthine oxidase (XOD) and adenosine deaminase (ADA) enzymes	[14,37]
	Upregulation of uric acid secretion transporters and downregulating uric acid reabsorption	[14,38]
Glucose metabolism	Inhibition of α-glucosidase, an enzyme that hydrolyzes disaccharide and oligosaccharides Increase glucagon-like peptide-1 for lowering blood glucose and improving glucose homeostasis	[39,40]
Insulin sensitivity	Restores phosphorylation of protein kinase B (AKT)/glycogen synthetase kinase and insulin receptor substrate-1 (IRS-1) through AMPK-activated pathway Restore and decrease ROS-induced pathway Stimulate glycogen synthesis	[41,42]
Hepatic gluconeogenesis	Phosphorylation of insulin receptor substrate-1 (IRS-1) and suppresses hepatic gluconeogenesis	[43,44]
Lipid metabolism	Increase serum glucagon-like peptide-1	[44]
	Reduction of total cholesterol Increase the HDL levels Reduce LDL cell uptake and inhibit LDL cholesterol oxidation	[36,45]
Free fatty acid oxidation	Activation of AMPK and upregulation of succinate dehydrogenase activity to maintain cell energy homeostasis	[43]
Anti-inflammatory	Interferes and suppresses activation of inflammatory nuclear factor kappa-B (NF-κB) and tumor necrosis factor-α (TNF-α)	[45,46]
Liver functioning	Lower expression of hepatic alanine aminotransaminase (ALT) and aspartate transaminase (AST), the hepatic enzymes indicative of liver injury	[46]
Gut microbiota	Targets gut microbiota by providing prebiotics function. Protects against the gut barrier Enhance the formation of beneficial short chain fatty acids (SCFAs) and amino acids	[47,48]
Bile acids metabolism	Targets bile acids metabolism, which mediates various metabolic pathways via G-protein-coupled receptor 1 and farnesoid X-activated receptor (FXR)	[49,50]

## Data Availability

Not applicable.
